# Virtual Reality Meditation for Fatigue in Persons With Rheumatoid Arthritis: Mixed Methods Pilot Study

**DOI:** 10.2196/46209

**Published:** 2023-10-17

**Authors:** Nathan J Dreesmann, Diana Buchanan, Hsin-Yi Jean Tang, Thomas Furness III, Hilaire Thompson

**Affiliations:** 1 Virtual Therapeutics Kirkland, WA United States; 2 School of Nursing Loma Linda University Loma Linda, CA United States; 3 Biobehavioral Nursing and Health Informatics School of Nursing University of Washington Seattle, WA United States; 4 Industrial and Systems Engineering College of Engineering University of Washington Seattle, WA United States

**Keywords:** anxiety, chronic pain, depression, fatigue, feasibility study, feasibility, head-mounted display, meditation, mixed method, mood, pain, rheumatoid arthritis, symptom, virtual reality, VR

## Abstract

**Background:**

Effective symptom management is crucial to enhancing the quality of life for individuals with chronic diseases. Health care has changed markedly over the past decade as immersive, stand-alone, and wearable technologies including virtual reality have become available. One chronic pain population that could benefit from such an intervention is individuals with rheumatoid arthritis (RA). Recent pharmacologic advances in the management of RA have led to a decrease in inflammatory symptoms (eg, chronic pain) or even disease remission, yet up to 70% of patients with RA still suffer from fatigue. While VR-delivered behavior, meditation, and biofeedback programs show promise for pain and anxiety management, there is little information on the use of virtual reality meditation (VRM) for fatigue management among individuals with RA.

**Objective:**

This study aims to (1) examine the feasibility of implementing a study protocol that uses VRM, (2) determine the acceptability of using VRM for fatigue management in an outpatient population, and (3) identify barriers and contextual factors that might impact VRM use for fatigue management in outpatients with RA.

**Methods:**

We used a convergent, mixed methods design and enrolled adults aged 18 years or older with a clinical diagnosis of RA. Patient-Reported Outcome Measure Information System (PROMIS) measures of fatigue, depression, anxiety, pain behavior, and physical function were assessed alongside the brief mood introspection scale at baseline and weekly for 4 weeks. VRM use across the 4-week study period was automatically stored on headsets and later extracted for analysis. Semistructured interview questions focused on feedback regarding the participant’s experience with RA, previous experience of fatigue, strategies participants use for fatigue management, and the participant’s experience using VRM and recommendations for future use.

**Results:**

A total of 13 participants completed this study. Most participants completed all study surveys and measures (11/13, 84% and 13/13, 100%, respectively) and were active participants in interviews at the beginning and end of the program. Participants used VRM an average of 8.9 (SD 8.5) times over the course of the 4-week program. Most participants enjoyed VRM, found it relaxing, or recommended its use (12/13, 92%), but 8 (62%) noted barriers and conceptual factors that impacted VRM use. On average, participants saw decreases in PROMIS fatigue (–6.4, SD 5.1), depression (–5.6, SD 5.7), anxiety (–4.5, SD 6), and pain behavior (–3.9, SD 5.3), and improvements in PROMIS physical function (1.5, SD 2.7) and Brief Mood Introspection Scale mood (5.3, SD 6.7) over the course of this 4-week study.

**Conclusions:**

While this study’s implementation was feasible, VRM’s acceptability as an adjunctive modality for symptom management in RA is contingent on effectively overcoming barriers to use and thoughtfully addressing the contextual factors of those with RA to ensure successful intervention deployment.

**Trial Registration:**

ClinicalTrials.gov NCT04804462; https://classic.clinicaltrials.gov/ct2/show/NCT04804462

## Introduction

Effective symptom management is crucial to enhancing the quality of life for those with chronic diseases. Health care has seen sweeping changes over the past decade as immersive, stand-alone, and wearable technologies like virtual reality (VR) have become available [1]. Most often deployed through a dedicated headset that blocks out external distractions, VR technology uses visual, auditory, and proprioceptive inputs to create a realistic, 3D environment that encourages a sense of physical presence in a virtual space [2]. These immersive, multisensory factors are thought to enhance VR’s therapeutic effects. As a therapeutic tool, various kinds of VR content have been developed and deployed to streamline mental health treatment and alleviate acute and chronic pain [3-5]. Rheumatoid arthritis (RA) is a debilitating chronic disease that affects over 1.3 million adults in the United States and nearly 35 million people worldwide [6,7]. Recent pharmacologic advances in the management of RA have led to a decrease in inflammatory symptoms (eg, chronic pain) or even disease remission [6], yet up to 70% of patients with RA still suffer from fatigue [8]. For those with RA, fatigue and pain management are closely tied together [9]. The most effective intervention for managing these symptoms tends to be cognitive behavioral therapies or movement-based approaches [9]. While VR-delivered behavior, meditation, and biofeedback programs show promise for pain and anxiety management [10,11], there is little information on the use of VR meditation (VRM) for fatigue management in those with RA.

The goal of this research study is to examine the feasibility and acceptability of using VRM to manage fatigue in outpatients with RA. The specific aims of this convergent mixed-methods study include (1) examining the feasibility of implementing a study protocol that uses VRM, (2) determining the acceptability of using VRM in an outpatient population, and (3) identifying barriers and contextual factors that might impact VRM use for fatigue management in outpatients with RA.

## Methods

### Design and Approach

**Figure 1:**
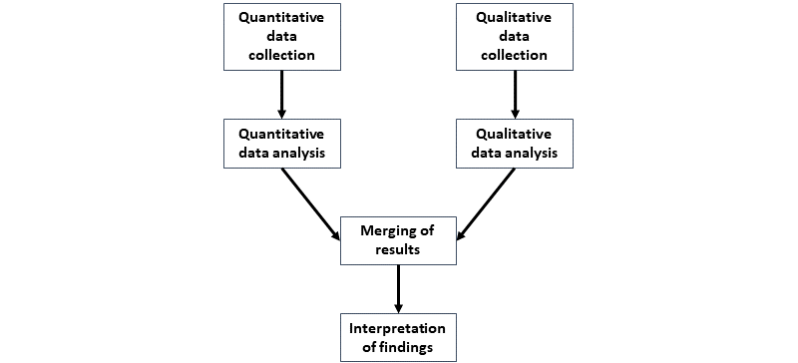
Convergent mixed methods design and analysis.

This study used a convergent, mixed methods study design, as detailed in Figure 1. Quantitative study data was collected and managed using Research Electronic Data Capture (REDCap; Vanderbilt University) tools hosted at the University of Washington’s Institute for Translational Health Services [12,13]. A qualitative thematic approach was taken to formulating questions and performing semistructured interviews with participants. For purposes of this study, feasibility (aim 1) will be evaluated by examining the completeness of study surveys and participation in pre- and poststudy interviews. Acceptability of the VRM intervention (aim 2) will be evaluated by examining VRM use during the study in conjunction with feedback from participants about their experience with the intervention. Lastly, barriers and contextual factors that might impact each of these first 2 aims will be explored through interviews at the end of the program (aim 3). Study design, methods, and approach were informed by symptom science experts (HT, DB, and JT) and a VR expert (TF).

### Recruitment

Eligible participants were aged 18 years or older with a diagnosis of RA. Exclusion criteria include individuals with a past medical history of uncorrectable visual or auditory impairment, a history of seizure disorder or seizure caused by technology use, extensive motion sickness, vestibular dysfunction, or excessive face or scalp sensitivity to pressure. These criteria were chosen because immersion and presence necessitate sufficient vision and hearing, technology use may cause side effects in persons with seizure history, severe motion sickness, or vestibular dysfunction, and scalp sensitivity could inhibit use of a VR headset. Recruitment occurred face-to-face at an outpatient rheumatology clinic before the COVID-19 pandemic, after which participants were recruited through posted flyers during the ongoing pandemic. The study team enrolled interested participants between November 1, 2019, and January 14, 2021.

### Patient-Reported Outcome Measures

This study uses the PROMIS (Patient-Reported Outcome Measurement Information System) measures banks for fatigue (v1.0), depression (v1.0), anxiety (v1.0), pain behavior (v1.0), and physical function (v1.2). The primary measure, fatigue, was chosen as it often persists even when RA (as a disease) is well managed [7,8]. Secondary measures of depression, anxiety, pain behavior, and physical function were selected based on their connection to fatigue and RA [9,10,14]. PROMIS scores range from 0 to 100 and use a T-score metric in which the mean of a relevant reference population is 50 (SD 10) [15]. On the T-score metric, a score of 40 is one SD lower than the mean of the reference population, and a score of 60 is one SD higher. PROMIS measures are scored using the T-score metric. Higher scores indicate more of the concept being measured. All PROMIS measures were deployed using their respective computer-adaptive test (CAT) forms. This method was chosen to ease participant burden and because PROMIS measures (especially fatigue, pain interference, and physical function) have been successfully validated in patients with RA [16]. The purpose of capturing these measures was to test study implementation and gain initial insight into the limited efficacy of the VRM intervention.

### Brief Mood Introspection Scale

At the end of the PROMIS CAT measures, participants completed the Brief Mood Introspection Scale (BMIS). The BMIS consists of 16 mood adjectives that range across predominantly positive or negative mood states; this study scored participants along the pleasant-unpleasant mood domain [17]. As above, the purpose of capturing these measures was to test study implementation and gain initial insight into the limited efficacy of the VRM intervention.

### Use Data

The date, time, and duration of VRM use were recorded for each participant. Use data was stored on headsets during use and extracted following the completion of the study. Time stamps and counts were corroborated with deidentified interview transcripts regarding VRM use to validate the data and ensure accuracy. It should be noted that while participants were given supporting materials regarding their headsets, they were given no direction regarding the frequency with which to use the VR headset so as not to influence participants’ natural use patterns.

### Semistructured Interviews

Semistructured interview questions focused on feedback about the participant’s experience with RA, previous experience of fatigue, and strategies participants use for fatigue management, as well as participant’s experience using VRM and recommendations for future use. Interviews were digitally recorded and transcribed verbatim. The semistructured interview guide (Multimedia Appendix 1) was adapted with input from symptom science experts (HT, DB, and JT) and a VR expert (TF). All interviews were performed in person (pre–COVID-19) or over the phone (during the COVID-19 pandemic) by the principal investigator (ND) or a member of the study team (Soothe Workgroup). Interviewers were asked to reflect on their own perspectives and thoughts (their “lens”) before and during each interview; these perspectives were noted at weekly meetings and during the analysis period.

### Procedures

Demographic and baseline data: following consent and enrollment, participants were emailed a survey link to complete demographic data (including age, sex, highest level of educational achievement, marital status, and employment status) and baseline PROMIS and BMIS measures. Before the COVID-19 pandemic and following the completion of baseline questionnaires, participants were interviewed in person before and immediately after their first VR session (which was observed). VR headsets were sent home with participants following their first session. Due to protocol changes required during the pandemic, participants were instead interviewed over the phone or through video chat, and conversations were digitally audio recorded. Following completion of their initial semistructured interview, headsets preloaded with VRM content and instructions for set-up, maintenance, and use were mailed to participants.

All participants were emailed links to complete PROMIS and BMIS measures on a weekly basis for a total of 4 weeks from the date they received their VR headsets. After completion of these questionnaires, a final semistructured interview was scheduled with participants in person (pre–COVID-19) or through phone or video chat (during the COVID-19 pandemic). VR headsets were collected during this final in-person meeting or mailed back to the study staff through a prepaid box and return label. Study completion was defined a priori as (1) receiving the VR headset, using it at least once, and returning it to the study staff, (2) completing at least one week’s worth of PROMIS and BMIS data in addition to the baseline measure, and (3) completing of 1 semistructured interview before and 1 semistructured interview after the intervention period. Missing data was addressed by using the last value carried forward, and all consent information as well as PROMIS, BMIS, use, and interview data were stored on a secure server at the University of Washington.

### VRM

To maintain safety and minimize the potential for VR side effects while using VRM, participants were given a training packet regarding VRM software and headset use. For their safety, participants were instructed to sit in a fixed chair with arm rests (without wheels or rollers). As a part of a prerelease program, VRM content (Virtual Therapeutics) was self-administered by participants. A freestanding and wireless Oculus Go (Facebook Technologies, LLC) was loaded with VR software content and used to deliver the intervention. When using VRM, users are immersed in a realistic 360-degree nature scene or built environment and guided through a meditative session by an audible voice. Sessions included meditation practices such as mindful breathing or relaxation techniques. Participants could turn their heads to look around the environment, but their “virtual selves” were fixed in position and could not move through the virtual space. During each session, participants chose their meditation practice and environment, as well as their session length (up to 15 minutes). There was no “masking” or “blinding” due to the obvious nature of wearing a VR headset. The VRM duration was limited to a maximum of 15 minutes based on the programmed content, which aligns with VR safety recommendations [18,19]. To better understand VRM use, participants were allowed to use VRM as frequently as desired, though they were instructed to have 1-hour “rests” between individual sessions to decrease the potential for VR side effects.

### Data Analysis

Descriptive statistics were performed for all variables, including demographics. For PROMIS measures, mean differences were calculated for each participant who completed the study by subtracting the baseline score from the average score (across 4 weeks of VRM use). As with PROMIS measures, mean differences were calculated for each participant who completed the study by subtracting the baseline score from the average score (across 4 weeks). All analyses were initially performed by the study team and reviewed by the principal investigator (ND). Interview transcripts were deidentified and reviewed for accuracy against recordings. Using a content-descriptive approach (open coding) [20,21], data were initially analyzed, data codes were inductively generated, and keywords were identified. A content analysis was then performed, and each transcript was coded line-by-line for first-level (descriptive) and pattern (theme) codes and cross-validated with another research team member. Atlas.ti v9 (ATLAS.ti Scientific Software) was used to support the management and coding of the data. Any conflicts in coding were resolved by group discussion and consensus. Individual reflexivity was addressed and discussed during the analysis period; the principal investigator (ND) and study staff were consistently encouraged to reflect on their perspectives and thoughts before and during data analysis.

Following the collection and analysis of PROMIS, BMIS, and VRM use data and the transcription and coding of semistructured interviews, the data were integrated to create a mixed methods matrix [22]. During the creation of this matrix, data were stratified based on VRM usage into categories of high, moderate, and minimal. Across these categories, mean (SD) and mean change scores were then calculated for all PROMIS and BMIS measures; mean (SD) use counts and use time were calculated, as well as the percentage of individuals who reported barriers to use, previous VR use, previous meditation experience, or symptoms of fatigue, pain, or sleep issues within these categories. This matrix allowed for an easier comparison of quantitative data with quantified, qualitative results of feedback from participants’ interviews [23-25]. Details on mixed methods design and analysis can be found in Figure 1.

### Ethical Considerations

Before the commencement of this study, its study protocol was reviewed and approved by the University of Washington’s institutional review board (approval number STUDY00007661). There were no monetary or nonmonetary incentives for participating in this study. Participation was voluntary; written informed consent was obtained from all participants before the COVID-19 pandemic, and electronic consent was obtained during the pandemic. All participant data was coded for analysis and then deidentified.

## Results

### Overview

**Figure 2:**
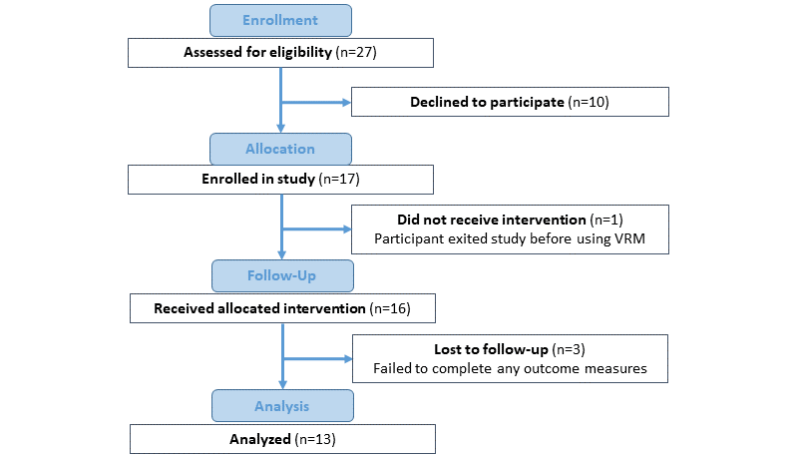
Study flow diagram. VRM: virtual reality meditation.

A total of 13 participants completed the study. Another 3 participants initially received the intervention but failed to complete any survey data and were lost to follow-up. Study flow details are noted in Figure 2. The mean age of participants was 52 (SD 16) years. They were primarily female (n=10), highly educated (bachelor’s, master’s, or professional degree; n=10), employed or retired (n=11), and had been in a marriage or domestic partnership at some point in their lives (n=11). Further demographic details can be found in Table 1. Based on interview data, 1 participant had a history of VRM use, and just under half (6/13, 46%) had a history of using meditation. All participants noted ongoing symptoms of both fatigue and pain during their interviews. During the study, a participant (1/13, 8%) noted negative side effects from VRM, and they discontinued use following this event (“I actually found myself getting a little motion sick, trying to use it” [Participant 11]).

**Table 1:** Demographic data.

Demographic		Value
Age, mean (SD)		52 (16)
**Gender, n (%)**		
	Female	10 (77)
	Male	3 (23)
**Education, n (%)**		
	Completed high school or GED^a^	1 (8)
	Some college	1 (8)
	Bachelor’s degree	3 (23)
	Master’s degree	5 (38)
	Professional degree	2 (15)
**Employment, n (%)**		
	Employed	9 (69)
	Unable to work	2 (15)
	Retired	1 (8)
	Self-employed	1 (8)
**Marital status, n (%)**		
	Single or never married	2 (15)
	Married or domestic partnership	5 (38)
	Divorced	4 (31)
	Separated	1 (8)
	Widowed	1 (8)

^a^GED: general educational development.

### Patient-Reported Outcome Measures

On average, participants saw decreases in fatigue (–6.4, SD 5.1), depression (–5.6, SD 5.7), anxiety (–4.5, SD 6), and pain behavior (–3.9, SD 5.3), as well as improvements in physical function (1.5, SD 2.7), and mood (5.3, SD 6.7) over the course of this 4-week study. Details on PROMIS scores can be found in Table S1 in Multimedia Appendix 2 and details on BMIS scores can be found in Table 2, respectively.

**Table 2:** BMIS Scores and use data.

Participant	BMIS^a^		Use data	
	Mean (SD)	Mean Δ	N	Time (minutes)
1	45.8 (6.5)	14.8	7	7.4
2	32 (6.8)	–6	3	6.8
3	30.5 (2.1)	5.5	4	9.6
4	38 (6.8)	–7	28	13.1
5	43.8 (13.5)	7.8	8	9.0
6	50.8 (2.2)	10.8	23	11.9
7	44 (0)	7	7	7.6
8	58.8 (7.5)	15.8	2	6.8
9	35 (3.6)	7	14	12.9
10	37.3 (1.7)	3.3	6	8.8
11	39.5 (5.9)	5.5	1	6.8
12	45.5 (1)	3.5	4^b^	—^c^
13	43.3 (3.6)	1.3	4	6.9
Overall	41.8 (7.7)	5.3 (6.7)	8.9 (8.5)^d^	8.9 (2.4)^d^

^a^BMIS: Brief Mood Introspection Scale.

^b^Based on patient reports.

^c^Not available.

^d^Mean (SD).

### Feasibility of Implementation

A total of 13 (13/17 76%) participants completed this feasibility and acceptability study (Table S1 in Multimedia Appendix 2 and Table 2). Out of which 11 participants (84%) completed all measures within the study and 2 (16%) failed to complete one or more weekly survey batteries. However, participants who began a weekly survey completed all measures for that time point. Overall, participants used VRM for an average of 8.9 (SD 8.5) sessions for an average duration of 8.9 (SD 2.4) minutes. A total of 3 participants used VRM for 14 or more sessions; another 3 participants used VRM for 7 or more sessions; and 6 participants used VRM for 6 or fewer sessions.

### Acceptability of Use

A total of 12 participants (92.3%) found VRM relaxing, enjoyed VRM, or recommended VRM use.

in the evening, it definitely helped better with turning some of that stress off and releasing some of the pressure that helps cause the fatigue

Participant 9

Patients appreciated having VRM to use in the moments that they needed symptom relief.

I really enjoyed VRM to tell you the truth…, because with [RA] it's cyclical…; some days are harder than others. And on a hard day actually having a meditation, like a VR meditation, would be really, really helpful

Participant 7

Most participants felt relaxed, or even energized, after using VRM.

It was relaxing enough to recoup. The batteries would recharge a little bit…, and it kind of invigorated [me]…, it woke me up. Yeah, it took me out of whatever funk I was in

Participant 3

After using VRM, a total of 5 participants (38.5%) reported better sleep, “It was wonderful…. [VRM] made me relax and… sleep better at night” [Participant 6]. A participant with previous experience using meditation noted that VRM assisted them to maintain their mindful focus:

I like the fact that [it’s] visual… [and there are] a lot of different ways they sort of [keep you] engaged with it…. Meditating in a quiet room…, your mind goes off in one direction [and]…. you got to remind yourself to come back to why you're sitting here…. That [VRM] system [makes it] …a little easier to stay on track.

Participant 4

Another participant noted that VRM allowed them to achieve “more general relaxation… than I feel I would have found through, just, you know, standard meditation without the VR” [Participant 9]. Each of these perspectives shows VRM’s acceptability as a tool for fatigue management in RA.

### Barriers to Use

Creating a conducive environment was essential to using VRM for symptom management, but a variety of barriers prevented participants from consistently using VRM. Over half of participants (8/13, 62%) noted barriers to consistent use of VRM: “I try to do it every… night before I [go] to bed. Sometimes I wasn’t able to…, it slipped my mind or [I] forgot…” [Participant 13]. Though VRM’s purpose was symptom management, sometimes these symptoms (eg, pain and fatigue) prevented VRM use.

…They're trying to change my… big biologic [medication] because it's not working very well anymore…. When I started with the VR headset, I was not feeling as bad as I am now. So for the first couple of weeks, it was really interesting and I enjoyed it…. But within two weeks, my hands really started hurting. And I couldn't use the trigger as well. But beyond that, when I'm in pain…, I found… I can't still my mind.

Participant 10

In addition to physical barriers, most participants were “not able to form any sort of daily habit of meditation.” [Participant 13]. Reasons for this included hardware and software issues.

I never paid attention to the types of errors… I just press the button on the side of the VR [headset] and turn it off and then start it again. And sometimes the little handheld [controller]… was a little screwy too

Participant 4

Issues with VR headset fit: “…[VRM] can be fun, but I didn’t really like the style of the headset and the fit and the comfort, so I didn’t use it very much” [Participant 12] and headset weight: “I had to look up a lot because of the heaviness of the headset” [Participant 12]. Participants also noted having trouble finding time for VRM.

It was hard finding times to do it, so I didn't do it all a whole lot, unfortunately. I wish I could have done it more, but I also didn't find myself like gravitating toward it…

Participant 2

Some participants noted having difficulty finding personal space to meditate.

I think part of what interrupted my usage is if I had other people around… [and] it just kind of felt awkward saying, ‘Excuse me, I'm going to go [meditate]’

Participant 1

Overuse was also an issue in this study. A total of 2 participants (15.4%) noted repeatedly using the headset for more than the recommended time in 1 sitting: “30 minutes maximum” [Participant 12], which proved too long for initial use.

I can see why you said no more than a half hour or whatever at a time.… [I would do] about two of…the longer [sessions]…. It went quickly, but it was, ‘when is this going to end?’

Participant 3

Both participants curtailed VRM use to a total of 4 times each during the study, and both noted significant barriers—dislike for elements of the VR environment and weight of the VR headset—that may be byproducts of extending sessions beyond the recommended duration. Thus, by engaging too quickly with longer VRM sessions, participants may have inadvertently created barriers to their use.

### Contextual Factors

Interviews uncovered contextual factors that may have impacted patient-reported outcome measures (PROMIS and BMIS) and VRM use. Participants noted issues with unclear, changing, and additional diagnoses: “So… vasculitis, RA, CHF and here I am” [Participant 3], inadequate symptom management*:* “I just finally hit the wall…. You stop, you have to. You can’t go on anymore because you can’t walk and every joint in your body hurts” [Participant 6], diffuse symptom sources: “I think it’s just [infuriating] not being able to understand that the source of where… my fatigue mainly stems from” [Participant 11], poor education about their health: “I still feel very unaware of all of the effects [of RA on] the day to day living, even though it's been two or three years now” [Participant 1], and frustration around uncertainty in their diagnosis:

It’s extremely frustrating, extremely frustrating…. There are so many factors in here…[that] it’s basically [the providers are] never sure what they're exactly dealing with

Participant 9

Socioeconomic status and living in a rural area heavily impacted participants access to care:

Out here is the physical location to services is horrible because even paratransit, trying to get, they won't… help pay for gas to go [to the clinic] because they said there's other rheumatologists that are closer. Well, the other rheumatologists that are closer were not accepting new patients that were on state medical. Things like that are huge barriers.

Participant 1

Participants had mixed feelings about the chronic care their RA required: “…when you have [a] disability you fight for [a] living” [Participant 8], and often placed a lot of pressure on themselves to manage their illness:

Yeah, I'm not very good at managing my [RA]. I mean having any sort of… illness is a commitment to take care of and I already have a lot of commitments in my life. So it's kind of just on the backburner a lot of the time

Participant 2

Some participants noted concerns about using VR: “…will I know what is reality and not, or will I want the virtual more than reality? So I guess that yeah, the line between reality and not, is what scares me” [Participant 11], yet others were drawn to the novelty of VR use, and some found a new tool for symptom management: “I do need to work on [meditation] becoming a habit” [Participant 1]. All participants noted being negatively impacted by the COVID-19 pandemic.

### Mixed Methods Matrix

During post hoc analysis and the creation of the mixed methods matrix, PROMIS and BMIS measures were categorized according to use counts of high- (≥14 times), moderate- (7-13 times), and minimal-use (1-6 times) groups (Table S2 in Multimedia Appendix 2). Post hoc categorization revealed decreases in fatigue, depression, anxiety, and pain behavior and increases in physical function and mood across all 3 use groups. Participants in high, moderate, and minimal groups used their headsets an average of 22 (SD 7.1), 7.3 (SD 0.6), and 3.3 (SD 1.8) times, respectively, over this 4-week study. While there was little difference in average use time between the minimal- and moderate-use groups, the high-use group used VRM for nearly twice the duration of time. Additionally, during interviews, a total of 1 (33.3%), 2 (66.7%), and 3 (42.9%) of the high-, moderate-, and minimal-use participants (respectively) reported active sleep issues related to RA, fatigue, and chronic pain. It should be noted that the 1 high-use participant who reported sleep issues before VRM use also reported drastic improvements after VRM use:

I always did it…before I went to bed and then by sleeping all night I wasn’t as tired during the day. …When I don’t use it, I’m really tired

Participant 6

## Discussion

### Overview

This study was hampered by the COVID-19 pandemic, barriers to use, as well as individuals’ lifestyles and contextual factors. Even so, the vast majority of participants were able to complete all study surveys and measures (11/13, 84% and 13/13, 100%, respectively) and were active participants in interviews at the beginning and end of the program. The first aim of this study was to determine the feasibility of implementation; the response from participants points to clear study feasibility. The second aim of this study was to determine the acceptability of VRM for fatigue management. While the approach may have been feasible, it is unclear if the VRM intervention was acceptable to participants based on their feedback and usage data. While most participants enjoyed VRM, found it relaxing, or recommended its use (12/13, 92%), on average, VRM was used less than twice a week (Table S1 in Multimedia Appendix 2). The third aim of this study was to identify what barriers or conceptual factors might impact VRM use. Subgroup analysis revealed that those who used VRM less also had a higher percentage of barriers to use (Table S2 in Multimedia Appendix 2); overall, 62% (8/13) of participants noted barriers and conceptual factors that impacted use. Barriers included issues with hardware and software, VR headset fit and weight, hand immobility (due to pain), as well as issues finding time and space for VRM. Similar problems are also reflected in studies by Meyer et al [26], Glegg and Levac [27], and da Cruz et al [28] that explore barriers to VR intervention implementation, use, engagement, and adherence. These barriers were compounded by the contextual factors that participants faced daily in managing their disease. The biopsychosocial impacts of fatigue and pain, paired with potential socioeconomic status and accessibility issues (eg, rural living), often left participants feeling exhausted and stuck. VRM provided a remote platform for participants to experience and practice meditation; while over half of participants noted some kind of barrier to use, nearly all had a positive experience with or enhanced symptom management from using VRM. This study also found that, on average, participants had decreases in fatigue, depression, anxiety, and pain behavior with increases in physical function and positive mood during this study. While this aligns with findings of VR’s (general) efficacy for managing anxiety, depression, fatigue, and pain [29,30], as well as emotions and mood [11], it should be interpreted cautiously as the sample size is small, SDs are large, and the majority of participants did not use the device consistently throughout the study. It should also be noted that 1 participant (8%) experienced mild, negative side effects from VR use: “a little motion sick[ness].” Similar rates of mild-to-moderate VR side effects (nausea related to “simulator sickness”) have been observed in other VR studies of symptom management [31,32].

Overall, these findings point out that while this study’s implementation was feasible, VRM’s acceptability as an adjunctive modality for symptom management in RA is contingent on effectively overcoming the barriers identified above and thoughtfully addressing the contextual factors of this population to ensure successful intervention deployment. Limitations to this study are primarily related to the occurrence of the COVID-19 pandemic after study commencement and its feasibility and acceptability. All aspects of this study were hampered by the COVID-19 pandemic, yet recruitment was severely impacted. This small recruitment limited the representation of this sample as it relates to the general RA population. Also, in this feasibility and acceptability study, there was no control group, which increases the potential for bias. As an emerging technology, VR-related side effects are a potential risk. Though participants were screened to minimize the potential for occurrence, a single participant did have mild side effects from VR use. As noted above, the small sample size, large SDs (for PROMIS and BMIS data), and inconsistent VRM use among participants somewhat diminished the generalizability of this study, but it did not detract from the achievement of study aims.

### Conclusions

This study adds to the current VR literature by providing key insights into barriers and contextual factors that impede VRM’s use for managing fatigue and associated symptoms in outpatients with RA. To overcome low usage of the intervention, coaching regarding consistent, daily use of the VRM, as well as education regarding VRM timing and duration of use, is recommended for successful intervention deployment. Improved instructions on the adjustment, application, and fit of VRM hardware are recommended for future studies. Additionally, the type of hardware being deployed for the VR intervention is paramount to its successful deployment. Careful consideration of the headset’s weight, how the weight is distributed on the head (is there a counterbalance?), and the duration of time users plan on engaging in the therapeutic activity will all impact the degree to which participants can consistently use the headset safely. Due to the reliance of patients with RA on medications for disease management and the potential fluctuation in disease course in RA, future intervention studies in this population should account for medication use, disease characteristics, and disease activities, especially “flare-ups” during the study period. Areas for future research include determining the efficacy of VRM for fatigue management, extending the duration of VRM use beyond 4 weeks, working with health care providers to study VRM use in other populations, and exploring how various kinds of VR-delivered meditation content might impact symptom management in these populations.

## Appendix

Multimedia Appendix 1 Interview guide.

Multimedia Appendix 2 Supplementary tables S1 and S2.

## Abbreviations

BMIS: Brief Mood Introspection Scale
CAT: computer-adaptive test
PROMIS: Patient-Reported Outcome Measure Information System
RA: rheumatoid arthritis
REDCap: Research Electronic Data Capture
VR: virtual reality
VRM: virtual reality meditation

## Data Availability

The data sets generated or analyzed during this study are available from the corresponding author on reasonable request.
