# Supplementary material for: Virtual Reality Meditation for Fatigue in Persons With Rheumatoid Arthritis: Mixed Methods Pilot Study
Source: JMIR Form Res. 2023 Oct 17;7:e46209. doi: 10.2196/46209 (PMC10618887; doi:10.2196/46209)
Supplement: Multimedia Appendix 1 [file formative_v7i1e46209_app1.docx]

## Part 1: Initial Interview

### Introduction

Thank you again for being willing to meet with us today. As you know I am a graduate student at University of Washington. I am interviewing you today as a part of a study focused on examining fatigue management in RA patients. We have read broadly about Rheumatoid Arthritis and how it can have many effects on physical functioning, mental health, and overall quality of life. With this in mind, it would be very helpful if we could learn about your everyday life and experience with RA – this should take about 30-45 minutes.

I am going to record this conversation to help us better remember your answers and allow a more accurate recall of how you describe your experiences – is that OK with you? {pause for answer}

Feel free to interrupt and ask questions at any time. If at any point you would like me to turn-off the recorder, or if there are any questions that you are uncomfortable with answering, please let me know.

1. Can you tell me a bit about your story of living with RA?/
   1. When were you diagnosed with RA?
   2. Tell me a bit about [year before diagnosis] [participant name]. What was he/she like?
2. Let’s go back to when you were first diagnosed with RA. Can you tell me more about what was going on during that time?
   1. *What were you feeling?*
   2. *You mentioned feeling [mention feelings]. Have these changed over time?*
3. Describe a typical day to me. What does a day in your life look like?

(LISTEN FOR: *lifestyle, active/non-active, responsibilities, daily tasks, sleep patterns, therapy/coping mechanisms*)

- 1. How has RA impacted your everyday life?
  2. Have you noticed any changes in your lifestyle?

1. What do you currently use to manage RA? Other than medication, have you used any alternative approaches to managing RA?
   1. Have you experienced any barriers to managing RA?
2. Have you tried meditation?
   1. *What was that experience like?*
   2. *Do you still actively meditate?*
   3. *Why did you stop? / What were some of the challenges you faced?*
3. When I say the word ‘fatigue’, what comes to your mind?
4. What role does fatigue play in your everyday life?
5. Many people living with a chronic disease like RA have aspects of their life that motivate them - what motivates you to manage living with RA?

## Part 2: During and After 1^st^ use of VR Meditation

### (onsite, pre-COVID-19)

1. How are you feeling today?
   1. *How would you describe your mood right now?*
   2. *How do you feel physically?*
   3. *Are you having any symptoms of RA that are particularly bothersome?*
2. When I say, “virtual reality”, what comes to mind?
   1. *Have you been in a virtual world/environment before?*
   2. *Tell me about your experience(s)*
3. Ok, I’m g*oing to have you put on the headset – I’m going to be wired into the headset so I can see what you’re seeing and guide you through using the headset for the first time*
4. Does it feel alright?
   1. How do you feel right now?
5. I’m going to have you select one of these meditation experiences. Would you like to try that? *<walk them through the setup>* Okay, I’m going to check in with you in a minute. Until then just try and relax.
6. (After) How would you describe your mood right now?
   1. How do you feel physically?
7. Tell me about your experience in VR.
   1. What was your favorite part?
   2. What were some of the challenges you experienced?
   3. *How does this compare to traditional meditation?*
8. Do you have any other questions about using the VR headset?

## Part 2: During COVID-19

### (continued from Part 1)

1. How are you feeling today?
   1. *How would you describe your mood right now?*
   2. *How do you feel physically?*
   3. *Are you having any symptoms of RA that are particularly bothersome?*
2. When I say, “virtual reality”, what comes to mind?
   1. *Have you been in a virtual world/environment before?*
   2. *Tell me about your experience(s)*
3. Do you have any other questions about using the VR headset?

## Part 3: Exit Interview

### Introduction

Thank you again for being willing to meet with us today. As you know I am a graduate student at University of Washington. I am interviewing you today as a part of a study focused on examining fatigue management in RA patients. We have read broadly about Rheumatoid Arthritis and how it can have many effects on physical functioning, mental health, and overall quality of life. With this in mind, it would be very helpful if we could learn about your everyday life and experience with RA – this should take about 30-45 minutes.

I am going to record this conversation to help us better remember your answers and allow a more accurate recall of how you describe your experiences – is that OK with you? {pause for answer}

Feel free to interrupt and ask questions at any time. If at any point you would like me to turn-off the recorder, or if there are any questions that you are uncomfortable with answering, please let me know.

1. How would you describe your experience with VR?
2. Were you able to make meditation a part of your daily routine?
   1. How frequently did you use the device?
3. How did it make you feel?
   1. Did you notice any lasting effects?
   2. How do you think it impacted the role fatigue plays in your life?
4. What were some of the challenges you experienced?
   1. What was your favorite part?
5. Is there anything that would have made using this VR Meditation system better for you?
6. How likely are you to recommend this experience to other patients with RA?
   1. Why?
