# Supplementary material for: Virtual Reality Meditation for Fatigue in Persons With Rheumatoid Arthritis: Mixed Methods Pilot Study
Source: JMIR Form Res. 2023 Oct 17;7:e46209. doi: 10.2196/46209 (PMC10618887; doi:10.2196/46209)
Supplement: Multimedia Appendix 2 [file formative_v7i1e46209_app2.docx]

**Table S1**. PROMIS (Patient Reported Outcome Measure Information System) scores.

| Participant | Fatigue | | Depression | | Anxiety | | Pain Behavior | | Physical Function | |
| --- | --- | --- | --- | --- | --- | --- | --- | --- | --- | --- |
|  | Mean (SD) | Mean Δ | Mean (SD) | Mean Δ | Mean (SD) | Mean Δ | Mean (SD) | Mean Δ | Mean (SD) | Mean Δ |
| 1 | 57 (7.6) | -9.1 | 57.4 (2.8) | -4.5 | 61.5 (4.8) | -8 | 58.4 (2.5) | -4.1 | 31 (1.6) | 0.1 |
| 2 | 65.8 (4.8) | -8.1 | 53.8 (8.1) | -7.2 | 66.4 (9.3) | -0.7 | 55.5 (3.3) | -6.2 | 38 (0.7) | 2.1 |
| 3 | 61 (2.7) | 1.9 | 60.6 (0.06) | -1.7 | 63.9 (2.4) | 2.6 | 58.7 (0.1) | -1.4 | 37 (0.9) | -2.6 |
| 4 | 59.2 (0.8) | 2.4 | 55.3 (3.6) | 1 | 55.8 (1.7) | 2.3 | 53.2 (1) | -2.4 | 57.3 (0) | 0.3 |
| 5 | 58.9 (3.7) | -3.9 | 52.5 (1.1) | -8 | 56.8 (5.6) | -4.7 | 60.2 (1) | -0.5 | 39.1 (0.8) | 0.5 |
| 6 | 53.2 (3.7) | -2.3 | 50.8 (4.6) | -4.6 | 53.7 (5.6) | -6 | 58.4 (1.3) | -1.7 | 39 (1.8) | 0.03 |
| 7 | 59.1 (0) | -9.7 | 51.3 (0) | -1.4 | 53.9 (0) | -7.6 | 60.6 (0) | -2.8 | 39 (0) | 7.6 |
| 8 | 40.5 (10.7) | -14.9 | 34.2 (0) | -20.1 | 36.5 (2.5) | -21.2 | 35.3 (0) | -20.5 | 43 (1.9) | 5.3 |
| 9 | 69.5 (2) | -4.4 | 62.6 (4.1) | -13.2 | 62.2 (2.2) | -3.4 | 57.7 (1) | -5.7 | 34 (0.8) | 1.5 |
| 10 | 60.2 (1.7) | -5.6 | 52.6 (1.9) | -6.3 | 58.7 (2.2) | -5 | 57 (1.8) | -0.4 | 35 (1.6) | -0.6 |
| 11 | 54.1 (3.4) | -11.8 | 59.2 (3) | -1.3 | 61.4 (4.3) | -1.9 | 59 (1.3) | -2.7 | 44.3 (1.1) | 0.6 |
| 12 | 56 (3.7) | -8 | 50.6 (1.4) | -3.7 | 54.5 (5.5) | -5.3 | 54.6 (1) | -1.7 | 43.8 (3.1) | 4.3 |
| 13 | 58.8 (5.2) | -9.5 | 56.7 (0.3) | -1.7 | 61.1 (1.7) | -0.2 | 57.1 (0.6) | -0.3 | 42.8 (2.1) | 0.3 |
| Overall | 58 (6.8) | -6.4 (5.1) | 53.7 (7) | -5.6 (5.7) | 57.4 (7.5) | -4.5 (6) | 55.8 (6.5) | -3.9 (5.3) | 40.2 (6.5) | 1.5 (2.7) |

**Table S2.** Patient reported outcomes, average use, and interview data categorized by usage.

| Use categories |  | High  (≥14 times) | | Moderate  (7-13 times) | | Minimal  (1-6 times) | |
| --- | --- | --- | --- | --- | --- | --- | --- |
|  |  | Mean (SD) | Mean Δ | Mean (SD) | Mean Δ | Mean (SD) | Mean Δ |
| PROMIS |  |  |  |  |  |  |  |
|  | Fatigue | 60.6 (8.2) | -1.4 (3.5) | 58.3 (1.1) | -7.6 (3.2) | 55.8 (8) | -8 (5.3) |
|  | Depression | 56.2 (6) | -5.6 (7.1) | 53.7 (3.2) | -4.6 (3.3) | 49.5 (8.8) | -6 (6.6) |
|  | Anxiety | 57.2 (4.5) | -2.4 (4.2) | 57.4 (3.8) | -6.7 (1.8) | 55.6 (10) | -4.5 (7.8) |
|  | Pain Behavior | 56.4 (2.8) | -3.2 (2.1) | 59.7 (1.2) | -2.5 (1.8) | 49.8 (8.3) | -4.7 (7.2) |
|  | Physical Function | 43.4 (12.3) | 0.6 (0.8) | 36.4 (4.7) | 2.7 (4.2) | 39.3 (3.8) | 1.3 (2.7) |
|  |  |  |  |  |  |  |  |
| BMIS |  | 41.3 (8.4) | 3.6 (9.4) | 44.5 (1.1) | 9.8 (4.3) | 40.4 (9.6) | 4.1 (6.5) |
|  |  |  |  |  |  |  |  |
| Use Data |  |  |  |  |  |  |  |
|  | N | 22 (7.1) |  | 7.3 (0.6) |  | 3.3 (1.8) |  |
|  | Time (min) | 12.6 (0.6) |  | 8 (0.9) |  | 7.6 (1.3) |  |
|  |  |  |  |  |  |  |  |
|  |  | (%) |  | (%) |  | (%) |  |
|  |  |  |  |  |  |  |  |
| Barriers to Use |  | 0 |  | 66.7 |  | 100 |  |
| Prior VR |  | 33.3 |  | 0 |  | 0 |  |
| Prior Meditation |  | 66.7 |  | 66.7 |  | 33.3 |  |
| Fatigue |  | 100 |  | 100 |  | 100 |  |
| Pain |  | 100 |  | 100 |  | 100 |  |
| Sleep Issues |  | 33.3 |  | 66.7 |  | 42.9 |  |
